# Improving Accuracy of Tomato Plant Disease Diagnosis Based on Deep Learning With Explicit Control of Hidden Classes

**DOI:** 10.3389/fpls.2021.682230

**Published:** 2021-12-16

**Authors:** Alvaro Fuentes, Sook Yoon, Mun Haeng Lee, Dong Sun Park

**Affiliations:** ^1^Department of Electronics Engineering, Jeonbuk National University, Jeonju, South Korea; ^2^Core Research Institute of Intelligent Robots, Jeonbuk National University, Jeonju, South Korea; ^3^Department of Computer Engineering, Mokpo National University, Muan, South Korea; ^4^Fruit Vegetable Research Institute, Chungnam A.R.E.S, Buyeo, South Korea

**Keywords:** deep learning, control classes, explicit control, target classes, tomato diseases and pests

## Abstract

Recognizing plant diseases is a major challenge in agriculture, and recent works based on deep learning have shown high efficiency in addressing problems directly related to this area. Nonetheless, weak performance has been observed when a model trained on a particular dataset is evaluated in new greenhouse environments. Therefore, in this work, we take a step towards these issues and present a strategy to improve model accuracy by applying techniques that can help refine the model’s generalization capability to deal with complex changes in new greenhouse environments. We propose a paradigm called “control to target classes.” The core of our approach is to train and validate a deep learning-based detector using target and control classes on images collected in various greenhouses. Then, we apply the generated features for testing the inference of the system on data from new greenhouse conditions where the goal is to detect target classes exclusively. Therefore, by having explicit control over inter- and intra-class variations, our model can distinguish data variations that make the system more robust when applied to new scenarios. Experiments demonstrate the effectiveness and efficiency of the proposed approach on our extended tomato plant diseases dataset with 14 classes, from which 5 are target classes and the rest are control classes. Our detector achieves a recognition rate of target classes of 93.37% mean average precision on the inference dataset. Finally, we believe that our study offers valuable guidelines for researchers working in plant disease recognition with complex input data.

## Introduction

Plant diseases and physiological disorders concern farmers and researchers as it directly impacts food security and, therefore, human well-being (Stewart and Roberts, 2012). Quantifying the impact of plant diseases on crops represents one of the most challenging problems in agriculture (Food and Agriculture Organization, 2006). Once a plant is infected, the damage can be easily propagated to the entire crop, causing several production and economic losses. Traditionally, crop monitoring is conducted by specialists in the field, which requires a higher level of expertise to understand the complexity of plants and their interactions with factors that cause plant anomalies. However, this task is often considered time-consuming, laborious, and prone to error since it involves human knowledge (Barbedo, 2018a). Therefore, earlier and automatic identification of plant diseases is required to support human labor as an efficient tool to monitor plants.

Following the success of deep neural networks (DNNs), mainly on large-scale image classification (Russakovsky et al., 2015) and object recognition tasks (Lin et al., 2014), over the last few years, several works have presented solutions to the problem of plant disease recognition in various crops. This technology has shown the potential to reduce negative impacts to the crop by promptly estimating the damage using non-intrusive sensors such as RGB cameras. Classification methods based on convolutional neural networks (CNNs) is the notation of convolutional neural networks. It should be separated from the reference (Mohanty et al., 2016) predict the type of disease using the features of the whole input image, and detection methods such as region-based recognition estimate both localization and classification using bounding boxes and confidence score respectively (Fuentes et al., 2020). In this line of research, our early work (Fuentes et al., 2017b) introduced a detector based on deep learning that automatically performs localization and diagnosis of 10 types of tomato plant diseases. Consecutively, we improved the recognition rate by introducing a refinement filter bank (Fuentes et al., 2018) to address the problem of false positives caused by the detector.

Encouraged by the results achieved by our previous works, we seek further improvements, especially to make the system more adaptable to new real-world greenhouse conditions (Barbedo, 2018b; Ferentinos, 2018). We are particularly interested in addressing the performance decay observed when a model is evaluated in new scenarios than those utilized for training. We believe, therefore, that there is still room to improve in this particular application and have identified the following causes: (1) The model is unable to generalize well in the presence of new data. For instance, when a system is exposed to limited information provided by datasets that are practically inadequate to cover the large variety of features. (2) Many of this information is new to the system and is often associated with one of the trained categories, leading to wrong predictions during inference. (3) Training data are hard to obtain and scarce. Still, it can also be severely affected by different visual appearances determined by the types of disease and infection stages, illumination, sizes, and background conditions.

In this research, we take a step towards the issues mentioned above and present an approach to improve model accuracy by applying a strategy that can help refine the model’s generalization capability. More specifically, we investigate the interaction between anomalies and their inter- and intra-class variations from the perspective of two categories: target classes and control classes. Based on that concept, our strategy works as follows: First, we utilize the target and control classes to train and validate a detector on images collected on a set of greenhouses (known data). Then, we apply the generated features for testing the system’s performance on an inference dataset (new data) where the goal is to specifically detect the target classes. Finally, our model becomes more robust during inference in new environments by explicitly controlling inter- and intra-class variations of the data utilized during training.

The contributions of our work are summarized as follows:

- We propose and explore a paradigm called “control to target classes” to improve the performance of our deep learning-based detector to deal with changes of new greenhouse conditions using target and control classes.
- Experimental results on our tomato plant diseases dataset show the efficiency of the proposed framework. We work on a more extended dataset than (Fuentes et al., 2017b, 2018) that includes more classes and samples and obtain a recognition rate of target classes of 93.37% mean average precision (mAP) during inference.
- From an information-theory perspective, we analyze the distribution of samples in the feature space using the t-SNE distribution (Maaten and Hinton, 2008) and confirm that our strategy can improve the generalization of target classes.
- We believe that our study can offer valuable guidelines for researchers working in domains of plant disease recognition with complex input data. Also, the potential of this technology aims to help farmers and non-expert people find problems associated with plant anomalies and diseases that affect crops.

The remainder of this paper is organized as follows: Section “Related Works” presents a review of related works and techniques for plant diseases recognition; Section “Materials and Methods” describes our proposed method; Section “Experimental Results and Discussion” shows the experiments and results; and finally, Section “Conclusion” concludes the paper and presents a discussion and guidelines for future works in the field.

## Related Works

In this section, we describe recent works related to our research. We introduce some baseline approaches on deep learning for image classification and object detection. Then, we review some techniques for plant disease recognition.

### Deep Learning Architectures

The massive accessibility of media and hardware technology has brought new opportunities for the application of deep learning into various research areas (Schmidhuber, 2015; Voulodimos et al., 2018). CNNs have become the leading method for feature extraction in the image classification task (Krizhevsky et al., 2012). State-of-the-Art CNNs include for instance, VGGNet (Simonyan and Zisserman, 2015), ResNet (He et al., 2016), and feature pyramid network (FPN; Lin et al., 2017). In contrast, object-based recognition focuses more on the individual regions containing objects than the whole image’s context (Szegedy et al., 2013). It addresses the problem by localizing and classifying multiple image regions containing objects using bounding boxes and confidence scores, respectively. In this regard, Faster R-CNN (FRCNN; Ren et al., 2016), SSD (Liu et al., 2016), and YOLO (Redmon et al., 2015) are commonly chosen as baseline meta-architectures for object detection due to their robustness and applicability. Furthermore, recent works have also focused on designing methods to improve the performance of DNNs using techniques such as data augmentation (Shorten and Khoshgoftaar, 2019), optimization (Le et al., 2011), normalization (Ioffe and Szegedy, 2015), transfer learning (Yosinski et al., 2014), network complexity (Livni et al., 2014), real-time processing (Choi et al., 2019), and training data (Johnson and Khoshgoftaar, 2019).

### Techniques for Plant Disease Recognition

In recent years, deep learning techniques have shown great efficiency in recognizing diseases and pests that affect plants. Thus, through its implementation, deep learning-based systems have become the leading technology to fulfill this task. Depending on the processing strategy, these methods can be divided into two categories: image-based disease classification and region-based disease recognition.

#### Image-Based Disease Classification

A breakthrough in the area is the work presented in Mohanty et al. (2016), where the authors used CNN architectures such as AlexNet (Krizhevsky et al., 2012) and GooogleNet (Szegedy et al., 2015) to categorize 26 diseases of 14 crop species. Although this method efficiently classified images containing diseases, its application is limited to using images collected in the laboratory with a single label and homogenous background. Similarly, Sladojevic et al. (2016) identified 13 types of diseases and healthy leaves using an AlexNet architecture with an average accuracy of 96.3%. They further applied various techniques such as data augmentation to increase the size of the dataset and fine-tuning with pre-trained networks on large-scale datasets to increase efficiency while training. In the same context, recent works extended the application to various types of crops such as tomato (Fuentes et al., 2017a; Liu and Wang, 2020), cassava (Ramcharan et al., 2017), grapes (Liu et al., 2020), and walnut (Anagnostis et al., 2020).

#### Region-Based Disease Recognition

In this category, our previous work (Fuentes et al., 2017b) on tomato plant disease recognition presented a robust and effective solution to provide more objective information such as the bounding box and confidence score. Consequently, to improve the results, we proposed a new technique (Fuentes et al., 2018), based on a refinement filter bank that mainly copes with the problems related to class imbalance and false positives. We exploited the detector’s capabilities to generate the corresponding regions of interest (ROIs) that contain the location and type of diseases and then used a CNN filter bank for verification of misclassified samples. We obtained a recognition rate of 96% through that implementation, which improved 13% over the results in Fuentes et al. (2017b).

Recently, region-based frameworks were extended to other crops and diseases. For instance, a method (Liu and Wang, 2020) to detect tomato gray leaf spots using a network based on YOLO-v3 (Redmon and Farhadi, 2018). Also, YOLO-v3 was used to detect goosegrass in strawberries and tomatoes (Sharpe et al., 2020). Another study (Afonso et al., 2020) applied deep learning for tomato fruit detection and counting in greenhouses. Furthermore, an application of region-based framework with sentence description was designed to characterize plant disease recognition using bounding box and text information (Fuentes et al., 2019).

### Data Availability for Plant Diseases Recognition

The availability of accessible data has also brought the opportunity to improve the accuracy of image-based disease classification approaches. A significant breakthrough is the Plant Village Dataset (Hughes and Salathé, 2015). Recent works used this dataset or part of it to validate their experiments (Mohanty et al., 2016). However, although this dataset created new opportunities for plant disease recognition, it presents several limitations to provide a natural characterization of the problem. Images are mainly collected in the laboratory and do not include conditions proper of real field scenarios. Also, single label images containing single leaves with homogeneous backgrounds do not show the actual situation where plants could be affected by multiple diseases not only in the leaves but also on other parts such as stems, flowers, and fruits. On the other hand, our dataset initially presented in our previous study (Fuentes et al., 2017b) provided a different way to overcome the problem by identifying both class and localization of diseases on images collected in real greenhouse scenarios, including complex background conditions.

The drawbacks of using data collected in the laboratory against images collected in the field are analyzed in Ferentinos (2018). In that work, the authors evaluated various CNN models to classify images of healthy and 58 distinct diseases from 25 different crops using both types of data, with the best accuracy of 99.53% using a VGG network. Although promising results showed the method’s utility, the success rate was significantly lower when using images collected in the field. Therefore, it demonstrated that image-based disease classification under actual field conditions is challenging because it includes more variations, especially in the background context.

Despite the availability of datasets, data is still scarce and hard to collect. Also, the desired performance is challenging to achieve uniformly for all classes since a system tends to prioritize classes with more samples while minimizing the contribution of the other classes. In this regards, a solution to the issues on data imbalance proposed to generate synthetic images using generative adversarial networks for image-to-image translation (Nazki et al., 2020). This strategy improved the learning process concerning the data distribution, reducing the class imbalance issues and shifting the decision boundary towards better performance.

## Materials and Methods

### System Overview

**Figure 1:**
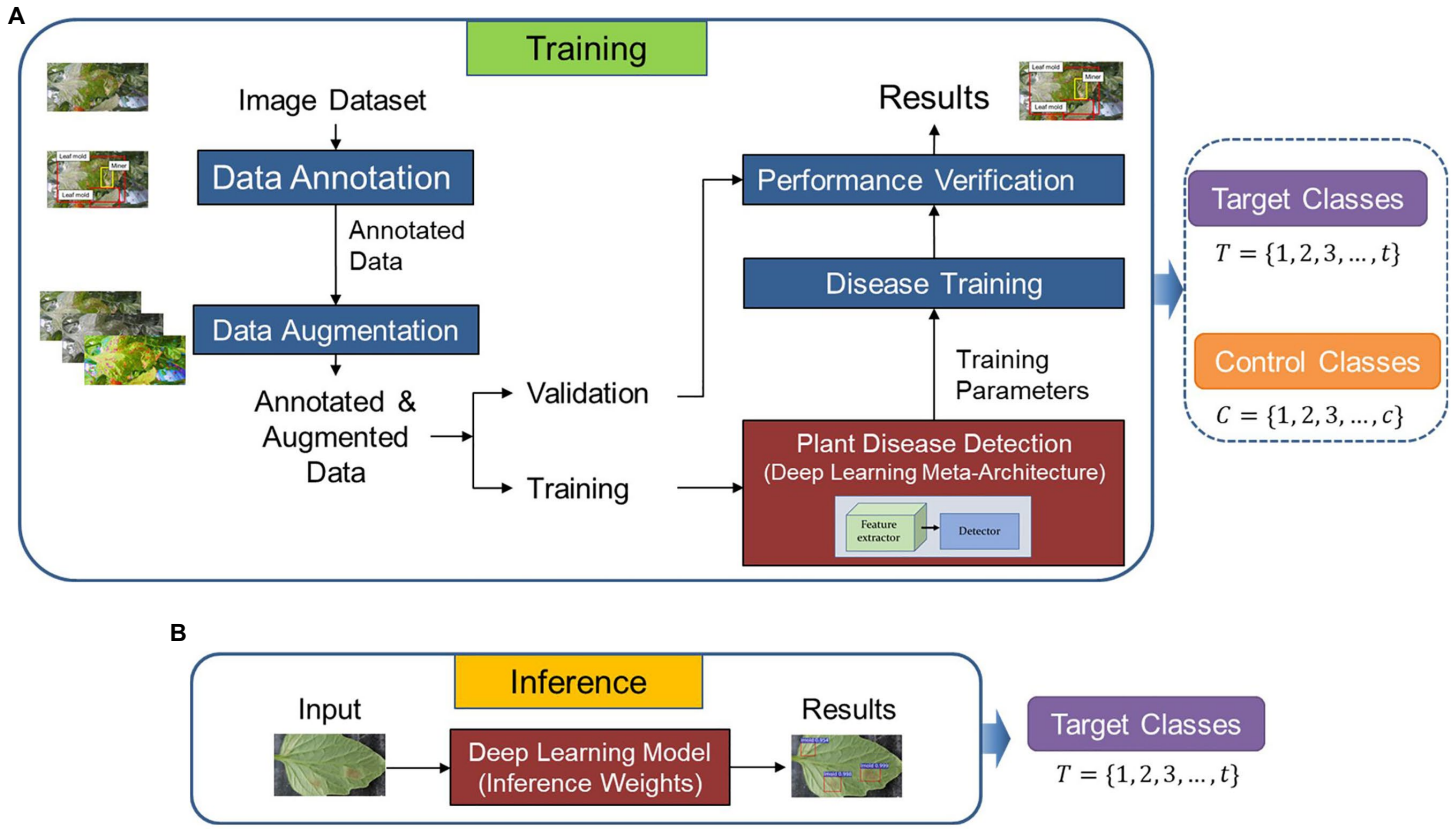
Overall architecture of the proposed method. **(A)** Training and validation. **(B)** Inference. Control and target classes are used for training and validation. The weights of the model are used during inference but only focusing on the target classes as the recognition goal.

Figure 1 presents the workflow of our proposed approach. The system operates as follows: First, we utilize a dataset of target and control classes to train and validate a detector on images collected in various greenhouses. Then, we apply the generated features for testing the inference of the system on data from other greenhouse environments to detect target classes exclusively.

In the following subsections, we study the conditions and strategies to achieve the desired performance through the recognition of tomato plant diseases. Each component of the system and the selection criteria for the target and control classes are described below.

### Criteria for Data Collection

Tomato plants, like any crop, are vulnerable to several physiological disorders and attacks caused by plant diseases. A plant is considered a bio-physiological organism and a physical object that is subject to physical laws (Geelen et al., 2018). Effective plant growth should be then based on balancing factors such as energy, water, and assimilates. A disequilibrium of those balances causes severe damages to the crop, for instance, due to abiotic disorders from environmental conditions such as temperature, humidity, air circulation, light, and plant species. In this sense, at indoor crops such as greenhouse cultivation, the conditions should be controlled to protect plants against external disturbances. However, the reality is that not all greenhouses count with appropriate technology to handle all variations. Many of the processes are still performed manually and demand the use of the farmers’ empiric knowledge or experts to decide a solution against a problem such as plant diseases. We studied those cases, and therefore, collected the dataset based on the following conditions:

- **Sensor:** We captured images using different RGB camera devices with various resolutions such as smartphones or other digital cameras, including DSLR cameras.
- **Images:** Our dataset includes images of multiple resolutions with various infection stages and locations of the symptom (mainly leaves, but also fruits and stems). Also, we collected images of healthy leaves and surrounding regions of the greenhouse.
- **Greenhouses:** We obtained data throughout the year since 2015–2020 in different seasons and local farms in Korea. The selected farms include some for research and commercial purposes at different scales. Among them, some utilized controlled environments and technology, while in others, mainly at a small scale, the process is performed more manually. Therefore, plants are more vulnerable to disease spread.
- **Diseases:** As tomato is a seasonal product, we visited the farms in coordination with local experts to ensure data collection of various diseases. The current dataset includes images of 12 types of diseases and pests in different amounts based on the presence and availability in the farms.
- **Time:** We collected data during the period of 10 am to 3 pm with sunlight.
- **Validation:** During the whole data collection process, we had the support of experts in plant diseases who were in charge of selecting and validating the type of diseases and disorders.

**Figure 2:**
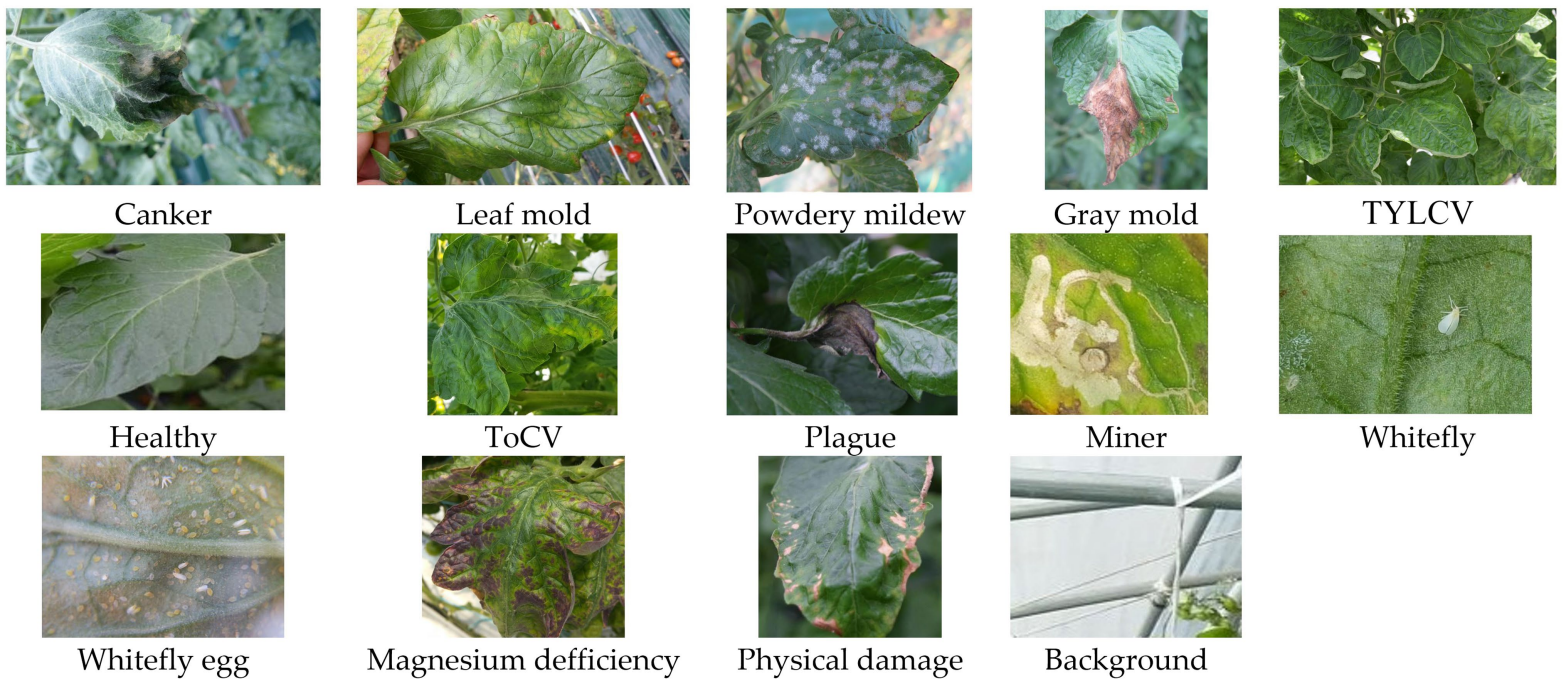
Example of collected images and types of diseases of our dataset.

Figure 2 shows an example of images and types of plant diseases and pests included in our dataset. A detailed description of the types of diseases and pests is presented in Fuentes et al. (2016). Hereinafter, we use the following notations in some of the tables and figures to represent the classes included in the dataset: canker, Canker; lmold, Leaf mold; powder, Powdery mildew; gmold, Gray mold; TYLCV, TYLCV/yculr; healthy, Healthy; ToCV, ToCV; plague, Plague; miner, Miner; wfly, Whitefly; wflyegg, Whitefly egg; magdef, Magnesium deficiency; phydam, Physical damage; back, Background.

### Target and Control Classes

**Figure 3:**
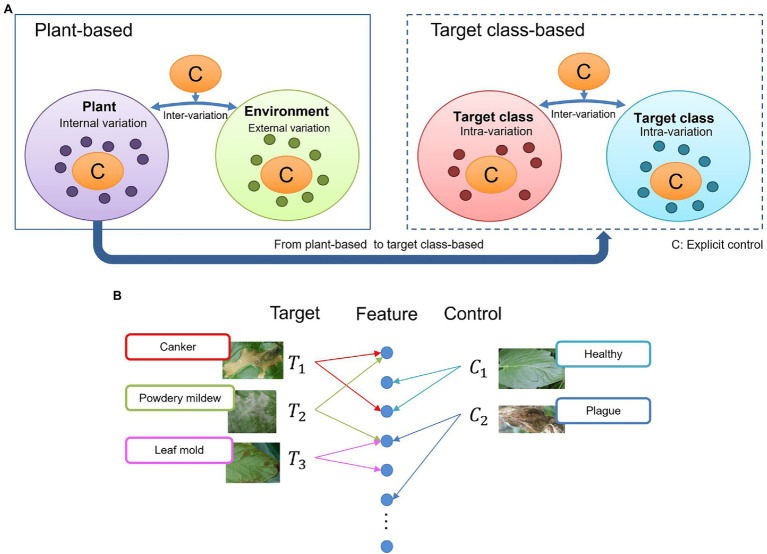
General overview of the context of the problem addressed by our proposed approach. **(A)** Intra-and inter class variations at plant-based and target class-based models. An explicit control of these variations can make a deep learning model become more oriented to learning various types of features. **(B)** Feature association in the space and feature domains. Similarities between diseases can affect the final prediction.

Target and control classes are selectable according to the given task. Initially, to find this distribution, we consider that once any disease infects a plant, the symptoms could appear in various parts such as leaves, stems, flowers, fruits. From the perspective of an image captured for recognition, these damages contain a universe of features showing several *internal variations* (Figure 3A, left). Also, various *external variations*, such as the lighting and surrounding objects in the greenhouse, can add complexity to the model. Some *intra-class variations* between diseases, may also appear, mainly if the infection occurs globally (e.g., whole leaf) or locally (e.g., leaf tip, spots). For instance, some diseases, especially at an early stage, contain features that can cause confusion to the system (Figure 3A, right).

Due to the large variety of data and infection stages in the feature domain, it is sometimes difficult for a system to associate images as part of the same distribution, resulting in wrong predictions and false positives (Figure 3B). For example, a leaf affected by powdery mildew can contain some features of canker since canker sometimes also appears at later stages of powdery mildew. Similarly, powdery mildew can cover some features related to leaf mold. Confusion can also be created by even unaffected parts of the leaves that show healthy regions or features of other diseases. From this assumption, we believe that by having *explicit control* over intra- and inter-class variations, a deep learning model can become more oriented to learning various types of features. Nonetheless, although these variations may not be part of the recognition goal, they still provide context information of the real scenarios. The system becomes then more robust as we reduce the chances of confusion.

Based on the above fundament, we considered the following conditions for selecting the target and control classes:

- **Target classes:** This group includes diseases that are mainly difficult to handle and demand a higher priority over the other classes. They spread faster and are challenging to be identified, especially at the early stage. Therefore, recognizing target classes is the main objective of our application. We selected five types of diseases as target classes for this study based on farms’ data availability and occurrence level. Control classes include: leaf mold, canker, gray mold, yellow leaf curl virus (TYLCV), and powdery mildew. We support this decision from the experience of our previous works and with the support of the experts.
- **Control classes:** Control classes are those that contain particular features that help deploy the system in new greenhouse scenarios. By using these classes, we aim to specifically obtain explicit control over intra- and inter-class variations by adding additional knowledge to improve the model’s generalization capability. Although the model also learns these classes, their application directly influences the final prediction of target classes. Control classes are healthy leaves, miner, physical damage, magnesium deficiency, tomato chlorosis virus (ToCV), plague, whitefly, whitefly egg, and background. The background class, in particular, provides contextual characteristics such as different illumination conditions and surrounding objects of the greenhouse.

### Deep Learning Meta-Architecture

Motivated by the above observations, this part elaborates the strategy in detail. As shown in Figure 1, the framework consists of two main parts: (1) training and validation, (2) inference.

#### Training/Validation Strategy

Following the promising results of our previous work (Fuentes et al., 2017b) with the FRCNN as the meta-architecture, we use it as the baseline model for our proposed approach. The FRCNN detector consists of a CNN backbone, a region proposal network (RPN) to obtain the object proposals, an ROI pooling layer, and fully connected layers followed by two branches for classification and bounding box regression. The RPN uses the features of the input image after being fed into the backbone CNN. For every point in the output feature map, the network should learn whether an object is included in the input image on its corresponding location and estimate its size. Next, the proposals from the RPN are used to pool features from the backbone feature map. This is done by the ROI pooling layer. The ROI pooling layer, in particular, works by taking the region corresponding to a proposal from the feature map; dividing this region into a fixed number of sub-windows, and performing max-pooling over these sub-windows to give a fixed size output. After passing these regions through two fully connected layers, the features are fed into the classification and bounding box regression branches.

Both target *T = {1, 2, 3, …, t}* and control classes *C = {1, 2, 3, …, c}* are used to build the weights of the baseline model. t and c represent the number of categories, respectively. Although the detection of controlled classes is not the priority of the system, they provide features and information of potential cases that could appear in greenhouse scenarios. On the other hand, target classes include those which are part of the recognition goal. Both groups are used during training/validation and testing on data collected in the seen farms.

Training the network end-to-end aims to reduce the final loss function in Equation (1), which adds the classification and regression losses. The objective is to reduce the loss between the predicted results and the ground truth, as well as to minimize the presence of false positives in the final results.


$$L\left(\left\{p_{i}\right\},\left\{t_{i}\right\}\right)=\frac{1}{N_{cls}}\underset{i}{\sum }L_{cls}\left(p_{i},p_{i}^{*}\right)+\lambda \frac{1}{N_{reg}}\underset{i}{\sum }p_{i}^{*}L_{reg}\left(t_{i},t_{i}^{*}\right)(1)$$

where $p_{i}$ and $p_{i}^{*}$ are the predicted probability of anchor i being an object and ground-truth label of whether i is an object, respectively, $t_{i}$ and $t_{i}^{*}$ are the predicted and ground-truth box coordinates, λ is a balancing hyperparameter. $N_{cls}$ and $N_{reg}$ represent normalization factors for classification and regression, respectively. Figure 1A shows the strategy for training and validation.

#### Inference

Once trained on data from seen farms, the model contains features from both target and control classes. Then, we use the generated weights to evaluate the adaptation capability of the model to new environments and its generalization to new data. This inference dataset includes samples of target classes collected in greenhouse environments other than those used for training. Control classes are omitted for recognition but still contribute the necessary weights to avoid class confusion and misclassification. Figure 1B shows the inference process.

### Evaluation Metric

Our system uses a single input image and generates a set of regions with bounding boxes and class confidence of plant diseases. We evaluate the performance of the detector using the following metrics:

- **Intersection-over-Union:** This metric evaluates the detector’s capacity to precisely localize the ROIs concerning the ground truth using the intersection over union (IoU) operation with a threshold value. We utilized a threshold of 50%.

$$IoU=\left|\frac{A\cap B}{A\cup B}\right|$$

(2)where *A* and *B* represent the ground-truth and predicted box, respectively.

- **Mean Average Precision:** mAP is the area under the precision–recall curve calculated for all classes.

$$mAP=\frac{1}{11}\underset{r\in \left[0,0.1,\ldots ,1\right]}{\sum }P_{interp}\left(r\right)$$

(3)


$$P_{interp}\left(r\right)=\underset{\tilde{r}:\tilde{r}\geq r}{\max}p\left(\tilde{r}\right)$$

(4) where, $P_{interp}\left(\tilde{r}\right)$ is the maximum precision for any recall values greater than *r*, and $p\left(\tilde{r}\right)$ is the measured precision at recall $\tilde{r}$ .

## Experimental Results and Discussion

In this section, we validate the performance of our proposed framework using target and control classes. We use the training/validation dataset to build the core features of the detector. Then, we evaluate the inference of the model with another set of target data from new greenhouses to perform recognition of target classes. Also, we further analyze the influence of control and target classes. Qualitative results show some examples of the output images of the detector evaluated in different scenarios. Finally, we demonstrate the impact of the use of target classes by representing features in the spatial domain.

### Dataset Settings

#### Training/Validation Dataset

Following our previous work (Fuentes et al., 2017b), we use the tomato diseases and pest dataset, including annotations for class and bounding box information. We apply geometric transformations (resizing, crop, rotation, horizontal flipping) and intensity transformations (contrast and brightness enhancement, color, noise) to augment the number of images in the dataset. Then, we divide the dataset into training and validation. The deep learning architecture uses the training dataset to obtain features of the regions containing diseases, and the validation dataset is used to validate the learning process during training. To facilitate our explanation, we will refer to this data as our “*baseline dataset*” and use it to build the core weights for further implementation.

Additionally, since our data come from different sources, an appropriate distribution is required to ensure that the system learns features adequately. Specifically, we apply an inner-class distribution of samples to capture data from all classes. This setup allows independent data from each class to appear during training and validation, respectively.

Table 1 shows the list of classes and the number of annotated bounding boxes obtained from approximately 10,000 images before and after data augmentation. This dataset includes 12 types of diseases and pests out of healthy leaves and an additional class containing background features. Five categories correspond to the target classes, and the rest are part of the control classes.

**Table 1:** Training/validation dataset of tomato plant diseases with target and control classes.

No.	Category	Class	Number of annotated samples^*^	Number of samples after data augmentation^*^
1	Target classes	Leaf mold	7,178	35,890
2		Gray mold	523	2,615
3		Canker	618	3,090
4		Powdery mildew	6,277	31,385
5		TYLCV	12,918	64,590
6	Control classes	Healthy	12,252	61,260
7		ToCV	4,190	20,950
8		Plague	598	2,990
9		Miner	2,328	11,640
10		Whitefly	1,701	8,505
11		Whitefly egg	6,314	31,570
12		Magnesium deficiency	584	2,920
13		Physical damage	1,767	5,835
14		Background	2,469	12,345
		Total	59,717	295,585

* Number of annotated bounding boxes obtained from approximately 10,000 images. In some cases, multiple diseases can be found in the same sample, therefore, we present the number of annotated boxes instead of the number of images for each class.

#### Inference Dataset

To further validate the use of target and control classes to improve our model’s generalization capability, we collected an inference dataset. We obtained additional data of target classes from farms other than those used for training. Although these sample images belong to the same type of diseases as the baseline dataset, their visual characteristics and background conditions may vary and contain more features from global and local areas of the leaves. In addition, we also extended the recognition of symptoms in other parts of the plants, such as fruits and flowers. Table 2 shows the number of images used for inference.

**Table 2:** Inference dataset of target diseases.

Class	No. images
TYLCV	134
Canker	487
Leaf mold	786
Gray mold	295
Powdery mildew	279
Total	1981

### Implementation Details

We conducted experiments on a machine with 4 NVIDIA TitanV GPUs, CUDA 9.0, and cuDNN 7.1.2 during the system development. We also implemented the model on a server PC equipped with an NVIDIA Tesla V100 GPU for inference purposes. For all the cases, we set the batch size to two images on a single GPU. We trained the model end-to-end using a pre-trained model on the MS-COCO dataset (Lin et al., 2014).

### Performance With/Without Explicit Control

#### Training/Validation on the Baseline Dataset

We train the model on the baseline dataset (Table 1) and evaluate the performance using the mAP. To prove the utility of control classes, we compare the model’s performance on two settings, first, training without control classes, and then with control classes. We use the same model in both cases, however, training without control classes represents the same configuration utilized in our previous work (Fuentes et al., 2017b). Moreover, we applied different feature extractors to find the most suitable for our approach.

Table 3 presents the results of this experiment. Using the exact configuration of our previous work (Fuentes et al., 2017b), with FRCNN as the meta-architecture and VGG-16 network as the feature extractor, without applying control classes reports a mAP of 87.06% for the validation on the target classes. Then, by adding the control classes to the training set, the performance improved by about 1.98%. Posteriorly, we replaced the backbone network with ResNet-50 and obtained a gain of 1.15% mAP using control classes. By further adding a FPN-based structure to ResNet-50, we improved the results at about 92.58% mAP, representing a gain of 2.8% to the results of the same model without control classes. In all cases, ResNet-50 FPN outperforms the other networks.

**Table 3:** Experimental results of training/validating the model on the baseline dataset with and without using control classes.

Class	VGG-16 (Simonyan and Zisserman, 2015)		ResNet-50 (He et al., 2016)		ResNet-50 FPN (Lin et al., 2017)	
	w/o control^*^	w/ control	w/o control^*^	w/ control	w/o control^*^	w/ control
Canker	0.8501	0.8737	0.8620	0.8951	0.8722	**0.9102**
Gray mold	0.8656	0.8806	0.8491	0.8700	0.9105	**0.9210**
Leaf mold	0.8973	0.9156	0.8801	0.9205	0.8904	**0.9360**
Powdery mildew	0.9083	0.9101	0.9027	0.9210	0.9358	**0.9419**
TYLCV	0.8317	0.8721	0.8732	0.9030	0.8801	**0.9200**
Total mAP	0.8706	0.8904	0.8734	0.9019	0.8978	**0.9258**

* w/o control represents the evaluation using the exact configuration of the baseline model (Fuentes et al., 2017b). Bold values represent the best results for each class.

**Figure 4:**
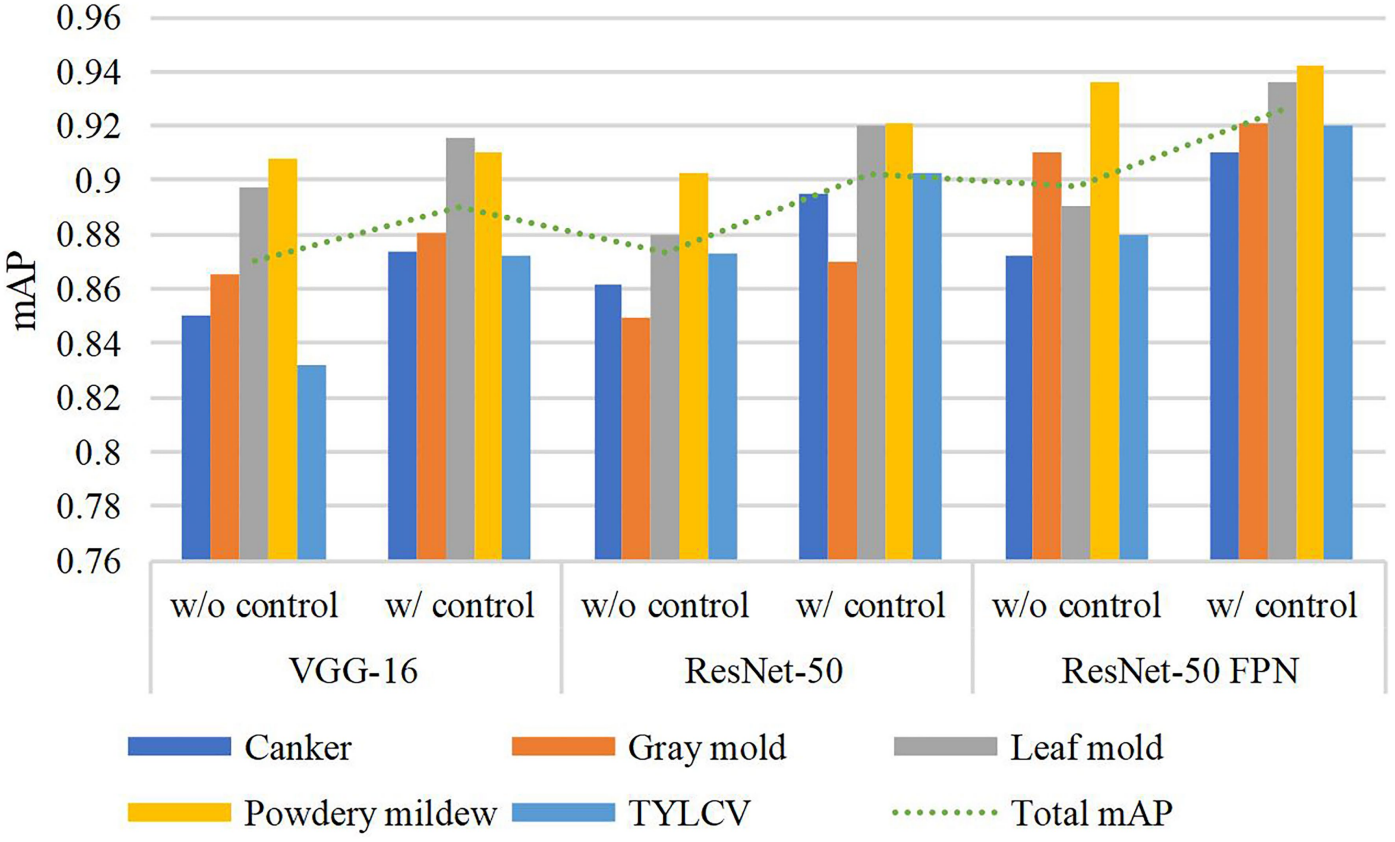
Performance differences with and without using control classes. A certain gain in mean average precision (mAP) is observed after adding the control classes along the target classes during training. Different feature extractors are evaluated in this graph.

**Figure 5:**
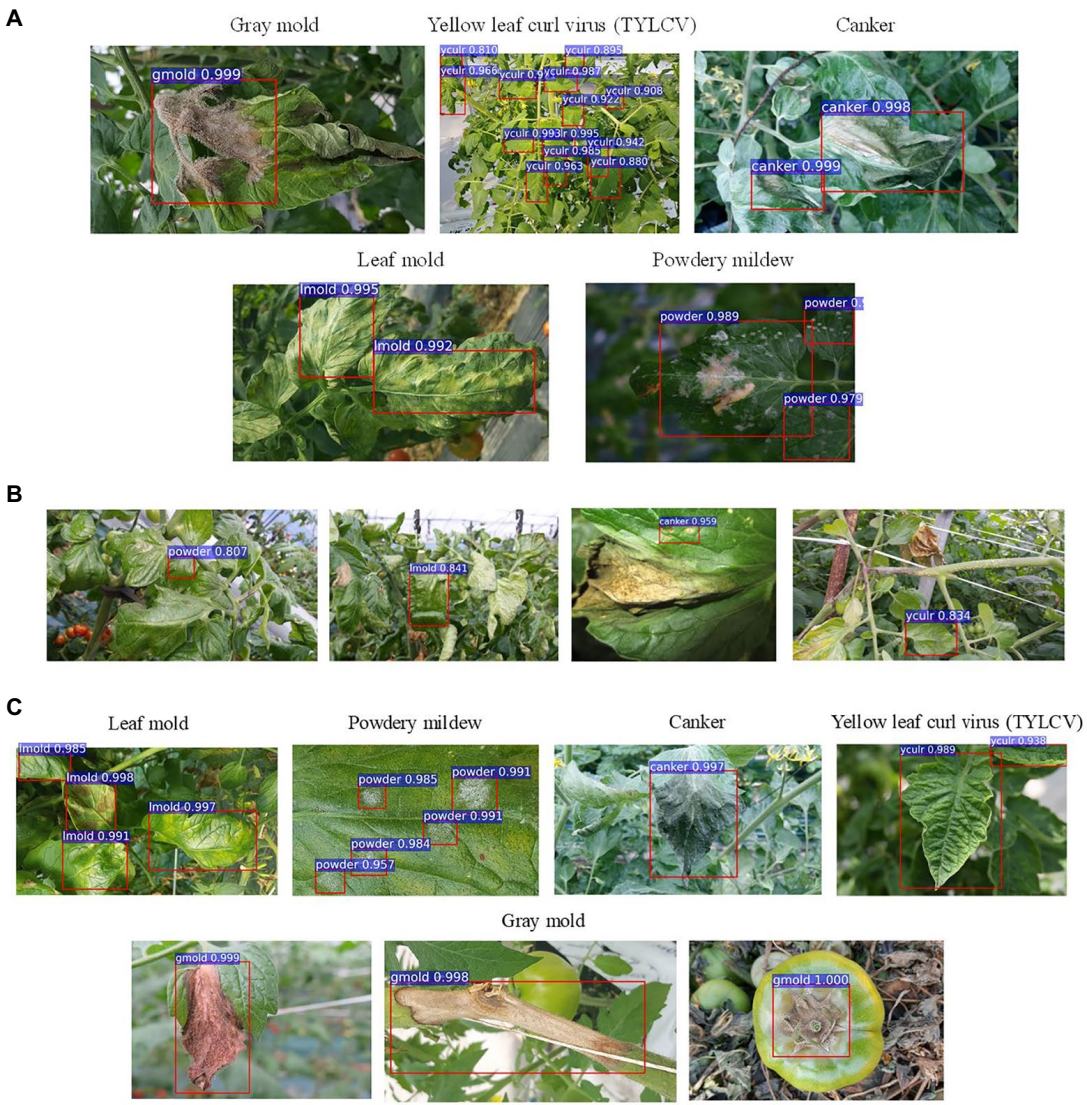
Example qualitative results from different setups and experiments. **(A)** Examples of true positives obtained during training/validation with target and control classes. **(B)** Examples of false positives and undetected areas resultant from training the model without control classes. **(C)** Recognized target samples on the inference dataset. Class notations are introduced in Section “Criteria for Data Collection.”

The use of control classes for training the model represents a valuable performance improvement for all the evaluated feature extractors. However, the results suggest that using an FPN-based architecture satisfactorily contributes to addressing the recognition problem of our approach. We believe that the reason is that FPN uses features obtained from different levels of the backbone and thus influences the recognition of objects at multiple scales. Figure 4 shows a representation of the performance differences after evaluating the model with and without control classes. Moreover, Figure 5A shows some qualitative examples of true positive results on images from the baseline dataset, and Figure 5B presents some examples of false-positives when training the model without control classes.

#### Inference on New Data

To measure the model’s capacity to deal with features of target classes, we further evaluate the trained model on the inference dataset using the best model of Table 3 (FRCNN ResNet-50 FPN). As shown in Table 4, despite the complexity of the inference data, our system can satisfactorily recognize an average of 93.37% mAP of target diseases. This result represents a difference of about 6.5% to the model trained without control classes. During inference, the system associates these features with the information obtained during training to improve the recognition capabilities of target classes. Therefore, we find that, by having explicit control over the intra- and inter-class variations, control data plays a crucial role in providing the required features to improve the recognition of target classes.

**Table 4:** Model evaluation of the inference dataset.

Class	w/o control	w/ control
Canker	0.8350	**0.9254**
Gray mold	0.8801	**0.9300**
Leaf mold	0.9087	**0.9520**
Powdery mildew	0.8532	**0.9211**
TYLCV	0.8641	**0.9402**
Total mAP	0.8682	**0.9337**

Bold values represent the best results for each class.

It is also essential to notice that the inference results show the potential characteristics of the proposed approach to deal with new conditions of target diseases. Specifically, healthy leaves and background classes support the model’s adaptation to new environments, while the other control classes add more context information. Figure 5C shows some qualitative example results of target disease recognition on the inference data.

### Discussion

#### Target Classes Over Control Classes

In this part, we study the inter-and intra-class variations that potentially help determine the correlations between target and control classes. Moreover, we evaluate the capabilities of the detector by studying the spatial distribution of features to demonstrate the contributions of our approach.

**Figure 6:**
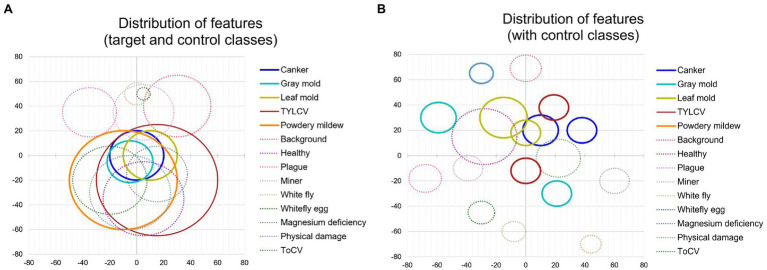
Distribution of features of target and control classes in the space domain. **(A)** Sampling distribution of the model trained without control classes. **(B)** Sampling distribution of the model trained with control classes. Each circle represents one class, and its radio depends on the number of samples and the dispersion of features. Solid lines and dashed lines correspond to target classes and control classes, respectively.

First, to show the importance of using control classes, we use the model trained on the baseline dataset (Table 1), applying only the target classes. We explore the features through the t-SNE distribution for all samples and visualize the relationship between classes. Then, we obtain the coordinates of the spatial location of each sample to generate Figure 6A for all classes in the dataset. Based on the information provided by this figure and using the criterion of region overlapping, we find that classes with a higher level of complexity are located mainly at the center of the distribution. Among them, we determine the target classes as those with significant inter-class variations such as canker, gray mold, and leaf mold. Still, more significantly, powdery mildew is a particular case that harms the system’s general performance if not treated appropriately. Correspondingly, in the case of the TYLCV, where symptoms appear more globally in the whole leaf area, the distribution is smoother as samples do not present such variations rather than sizes and infection stages. Yet, the area is larger, and generally, its features cover most of the classes. On the other side, concerning control classes, except miner, whitefly, whitefly egg, and background, the rest are covered either by other control classes or target classes, respectively.

Additionally, to demonstrate the effectiveness of using control classes for training, in Figure 6B, we present the case when both target and control classes are used for training. We can see that explicit control over the inter-and intra-class variations significantly helps the network avoid class confusion. Samples are then recognized as their corresponding categories and tend to make distant groups in the space. We extend this comparison in the next part.

#### Quality of Control Data

To support the results presented above, we evaluate the generated models in terms of the number of true positives (correct predictions) compared to the false positives and false negatives. In this experiment, we also analyze the capability of the system to quantitatively predict target classes and their impact on the false positive and false negative rates, using two cases:

- **Training without control classes:** We train the model on the target classes and further evaluate it in the whole dataset. In this setting, the system does not receive any features of the control classes.
- **Training on both target and control classes:** We evaluate the impact of adding the control classes to train the model along with the target classes. In this case, the system obtains features of both groups.

**Figure 7:**
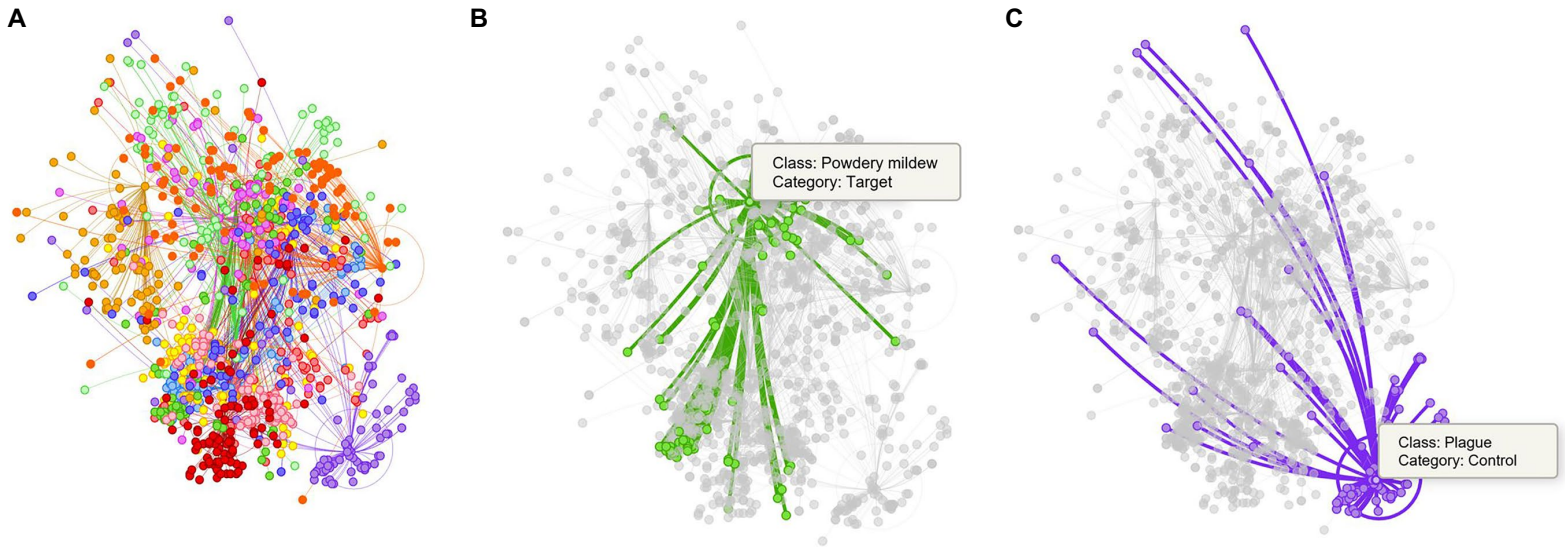
Representation of the tSNE distribution obtained from the model trained without control classes. **(A)** t-SNE distribution for the whole dataset. **(B)** Powdery mildew (target class) and its corresponding samples in the features space. **(C)** Plague (target class) and its samples in the space. Each dot represents a sample, and the colors identify the class assignation. Lines between dots show the connection of each sample to the center of its corresponding class.

In the first case, as presented in Table 5, target classes are effectively identified with slight levels of confusion between them. However, when evaluating the model on the rest of the dataset, depending on the class, higher levels of confusion mainly appear, for instance, with healthy, plague, and in lower amount with physical damage. In general, without control data used for training, the results evidence a total of 42.2% confusion. To support this statement, we obtain the t-SNE distribution, as presented in Figure 7, to find the location and class of the evaluated samples in the feature space. This representation evidences the model’s generalization problem to deal with new data. Testing on new classes generally diminishes the recognition of the target classes as they tend to confuse the network. We associate this scenario as an effect of the inter-and intra-class variations.

**Table 5:** Confusion matrix of target classes without using control classes for training.

Target classes*						Control classes*									Amount of confusion (target w/o control)
	lmold	gmold	canker	powder	TYLCV	healthy	ToCV	plague	miner	wfly	wflyegg	magdef	phydam	back	
lmold	90.0%	1.0%		2.0%		1.0%		4.5%				1.0%	0.5%		7.0%
gmold	2.0%	85.0%		2.5%		2.0%	0.5%	5.0%					3.0%		10.5%
canker	1.0%	0.5%	87.3%					6.6%	0.4%				4.2%		11.2%
powder	1.5%		1.0%	92.0%	3.0%	2.0%		0.5%							2.5%
TYLC V					89.0%	5.0%	3.0%		2.0%			1.0%			11.0%
Total confusion															42.2%

^*^Class notations are introduced in Section Criteria for Data Collection.

**Figure 8:**
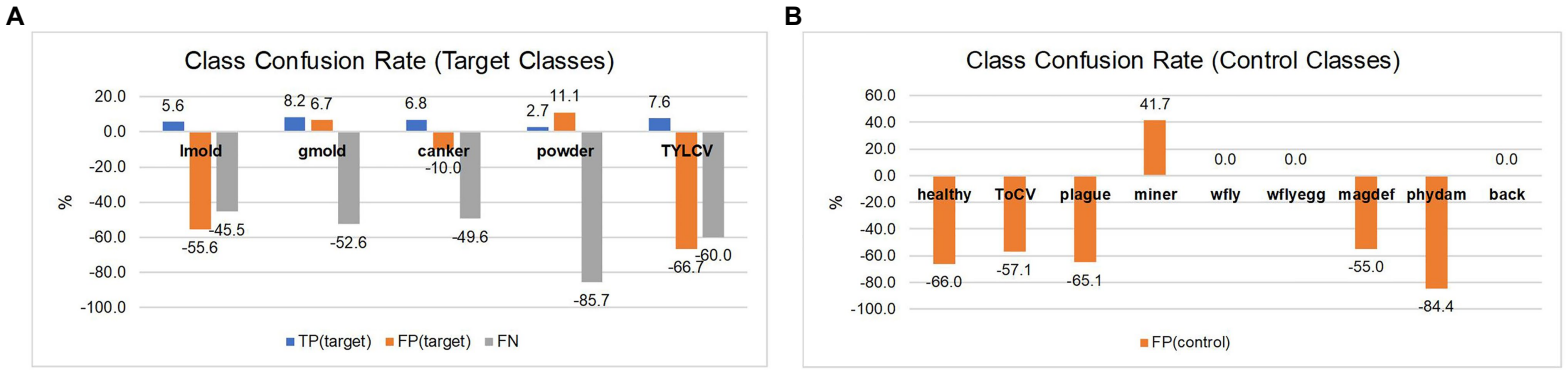
Class confusion rates concerning the use of target and control classes. **(A)** Using control classes can effectively reduce the false positive and false negative rates in most target classes. **(B)** Also, the effect is extended to reduce confusion with data of control classes mainly. The impact appears to be more effective in classes with more similar features to the target ones. Class notations are introduced in Section “Criteria for Data Collection.”

In the second case, as shown in Table 6, we can see a general tendency of improvement after adding control classes for training. The level of confusion decreased by about half to 19%. More importantly, by introducing control data during training, the number of true positives for the target classes increased, consequently benefiting the model for further applications in new farms. Also, it shows a significant reduction in the false positive and false-negative rates. Figure 8 illustrates the changes in confusion rates for the target and control classes.

**Table 6:** Confusion matrix of target classes using control classes for training.

Target classes*						Control classes*									Amount of confusion (target w/ control)
	lmold	gmold	canker	powder	TYLCV	healthy	ToCV	plague	miner	wfly	wflyegg	magdef	phydam	back	
lmold	95.0%	1.0%		1.5%				2.0%					0.5%		2.5%
gmold	1.0%	92.0%	0.4%	1.5%		0.8%		2.3%	1.5%				0.5%		5.1%
canker		0.6%	93.2%	2.0%			0.5%	1.5%	0.2%				2.0%		4.2%
powder	1.0%		0.5%	94.5%	1.0%	0.5%			1.0%			0.5%		1.0%	3.0%
TYLC V					95.8%	2.1%	1.0%		0.7%			0.4%			4.2%
Total confusion															19.0%

^*^Class notations are introduced in Section Criteria for Data Collection.

**Figure 9:**
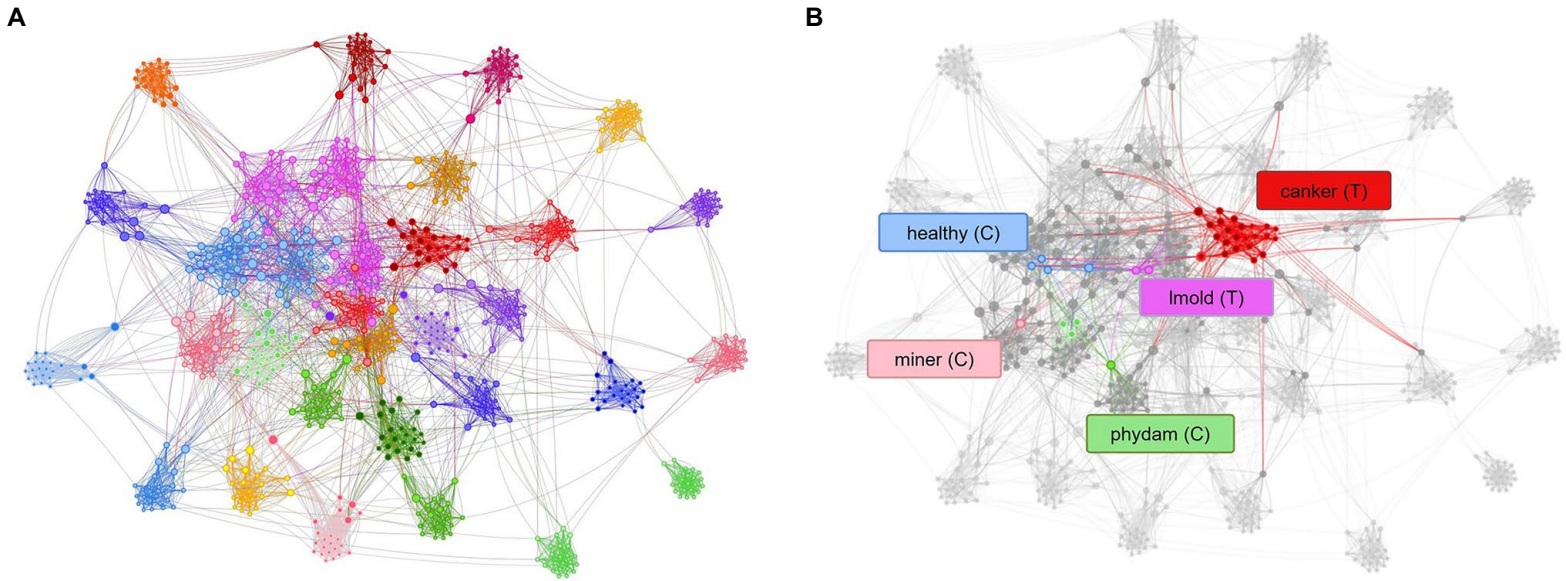
Representation of the tSNE distribution obtained from the model trained using control classes. **(A)** t-SNE distribution and class association. **(B)** Control classes provide sufficient information to assign samples to their respective category. Each dot represents a sample, and the colors identify the class assignation. Lines between dots show the connection of each sample to the center of its corresponding class. Class notations are introduced in Section “Criteria for Data Collection.”

Figure 9A shows the t-SNE distribution obtained after training the model with control classes. The generalization capability of the system improved, and samples appear to make groups that specifically occupy a region of the space. Furthermore, the confusion levels were reduced while the performance of target classes increased. Figure 9B shows the case when a set of samples are assigned to their corresponding classes. This result suggests that teaching the model with additional information from intra- and inter-class variations helps improve the recognition of target classes while reducing the presence of misclassified samples in the final prediction.

#### Current Limitations

Despite the satisfactory and robust performance that we presented, there is a limitation of the proposed approach. The main limitation is the data imbalance. This issue directly impacts the selection of the target classes as the recognition objective of a system. Data should be sufficient to capture all features that the system can encounter in real-world greenhouse scenarios. This fact indicates that while we can achieve satisfactory results on the evaluated target classes, the promising model still needs more data to improve its robustness against more variations. Also, an appropriate selection of samples is essential for the success of our approach.

## Conclusion

In this paper, we proposed a new paradigm called “control to target classes” to refine the generalization capacity of plant disease recognition based on deep learning. We presented a strategy to deal with changes in new greenhouse conditions. The explicit control over inter-and intra-class variations allowed our model to learn more data variations that make the system more adaptable and robust when applied to new scenarios. Experimental results on our extended tomato plant diseases dataset with 5 target classes and 9 control classes validated the performance of the proposed framework. We obtained a recognition rate of 93.37% mAP for the target classes during inference. From an information-theory perspective, we analyzed the distribution of samples in the feature space using the tSNE distribution. We confirmed that our methodology using control classes improved the recognition of target classes. Finally, our study can offer valuable guidelines for researchers working in plant disease recognition with complex input data. Also, the potential of this technology can help farmers and non-expert people find problems associated with plant anomalies and diseases that affect crops. Future studies will apply our proposed method to other crops using data collected in more greenhouse settings.

## Data Availability Statement

The datasets presented in this article are not readily available because this research has been carried out as part of a project funded by the Government of Korea. We have an agreement not to open the dataset until the project is concluded. Requests to access the datasets should be directed to AF, afuentes@jbnu.ac.kr.

## Author Contributions

AF designed the system, performed the experiments, and wrote the article. DP and SY advised in the design of the system and analysed the strategies to find the best method for plant disease recognition. ML collaborated and advised during data collection and annotation. All authors contributed to the article and approved the submitted version.

## Funding

This research was supported by the Basic Science Research Program through the National Research Foundation of Korea (NRF) funded by the Ministry of Education (No.2019R1A6A1A09031717); by the National Research Foundation of Korea (NRF) grant funded by the Korea government (MSIT) (NRF-2021R1A2C1012174); and by Korea Institute of Planning and Evaluation for Technology in Food, Agriculture and Forestry (IPET) and Korea Smart Farm R&D Foundation (KosFarm) through Smart Farm Innovation Technology Development Program, funded by Ministry of Agriculture, Food and Rural Affairs (MAFRA) and Ministry of Science and ICT (MSIT), Rural Development Administration (RDA) (421005-04).

## Conflict of Interest

The authors declare that the research was conducted in the absence of any commercial or financial relationships that could be construed as a potential conflict of interest.

## Publisher’s Note

All claims expressed in this article are solely those of the authors and do not necessarily represent those of their affiliated organizations, or those of the publisher, the editors and the reviewers. Any product that may be evaluated in this article, or claim that may be made by its manufacturer, is not guaranteed or endorsed by the publisher.
